# Safety Practices and Perceptions Among Laboratory Employees at a Tertiary Care Hospital

**DOI:** 10.7759/cureus.73245

**Published:** 2024-11-07

**Authors:** Lohith Kumar, Balaji Radhakrishnan, Mohammad Khalith, Varaprasad Mateti Devi, Anantharaman Venkatraman Vaidya, Meenakshisundaram Senthilnathan, Jano Roy Sellam George, Shivashekar Ganapathy, Bhuvanamha Devi Ramamurthy

**Affiliations:** 1 Pathology and Laboratory Medicine, Sri Ramaswamy Memorial Medical College Hospital and Research Centre, Sri Ramaswamy Memorial (SRM) Institute of Science and Technology, Chennai, IND; 2 Community Medicine, Government Medical College, Madanapalle, IND; 3 Community Medicine, Sri Ramaswamy Memorial Medical College Hospital and Research Centre, Sri Ramaswamy Memorial (SRM) Institute of Science and Technology, Chennai, IND; 4 Pathology, Mahatma Gandhi Medical College and Research Institute, Pondicherry, IND; 5 Pathology, Faculty of Medicine, Sri Ramaswamy Memorial Medical College Hospital and Research Centre, Sri Ramaswamy Memorial (SRM) Institute of Science and Technology, Chennai, IND

**Keywords:** air conditioning system, gloves, laboratory employee, noise, safety perception, safety practice

## Abstract

Introduction: Clinical laboratories form an important link in the healthcare chain. Raising the safety standards is an important aspect of the transformation of an organizational culture to provide good-quality healthcare services. The present study aims to determine the safety practices and perceptions of the existing system of safety among laboratory employees.

Materials and methods: This observational cross-sectional study was carried out among laboratory healthcare workers at a tertiary care hospital in Tamil Nadu, India. A pilot study (10% of the sample size) was also done to assess reliability. The sample size is 150: n = Z²pq / d², where Z = 1.96, p = 38.7, q = 61.3, and d = 7.7. Substituting the values in the formula, we have: n = [(1.96)² x 38.7 x 61.3] / (7.7)² = 150. The study was conducted using a questionnaire to gather demographic and professional data and details regarding the safety practices and perceptions of the existing system of safety among laboratory employees. Statistical analysis was done using Microsoft Excel 2010 (Microsoft Corp., Redmond, WA) and IBM SPSS Statistics for Windows, Version 23.0 (Released 2015; IBM Corp., Armonk, New York, United States).

Results: In this study, there were 99 female and 51 male participants. In the safety practices among laboratory technicians, 62% (n = 93) noted that they do not wash their hands before wearing gloves and 20% (n = 30) consume food and beverages during work hours. In the responses to perceptions related to job responsibilities, 50.7% (n = 76) of the lab technicians consider that noise in the workplace causes a disturbance, and 47.3% (n = 71) consider that infrequent cleaning of air conditioners causes health issues. The rest of the items in the questionnaire have scores between 80 and 100%. Safety practices in relation to safety perceptions were statistically significant with respect to age, overall safety perception, job responsibilities, and communication.

Conclusion: Overall, lab employees have high awareness and good adherence to safety practices. The current study concludes that a regular assessment of the safety climate is one way to enhance the organizational culture, motivate the workforce, and improve the quality of health services because it enables the identification of the aspects that require more attention and collaboration on quality to create a safety culture that encourages continuous improvement.

## Introduction

Safety is one of the factors that affect the quality of healthcare services. To maintain and enhance safety practices and deliver high-quality healthcare services, it is necessary to regularly assess the safety climate [1]. A poor safety culture is associated with unfavorable incidents caused by an unstable work environment, while a positive safety culture encourages continuous improvement [2].

Worldwide, only significant laboratory mishaps are documented, while near-misses including fires, chemical leaks/spills, glassware explosions, and equipment failure due to misuse are underreported [3,4]. The Occupational Safety and Health Administration (OSHA) in the United States and other national safety boards had previously gathered and examined data on lab incidents as a whole. Furthermore, not all institutions or laboratory staff employed by universities comply with the OSHA requirements [5]. As a result, there are inconsistencies in routine laboratory safety practices. Unfortunately, the factors that contribute to lab accidents have not been extensively studied. Many contributing factors are conceived at various levels, including individuals (knowledge, experience, choices of research staff conducting the study, characteristics or qualities of the principal investigator), laboratories (risks related to materials or equipment, skills, knowledge), departments (inadequacy), time management, indifferences, lack of awareness of safety requirements, lack of leadership, and lack of focus on regulatory compliance, institutions, and systems [6-8]. The “Swiss cheese” model of accident causation, according to researchers in the broader field of occupational safety, proposes that accidents are most likely to occur when several faulty parts of the system align. Even with education, “the human behavior component proved the toughest to manage of all the variables in accident prevention” [9-11]. Although awareness was high, previous studies revealed that there were shortcomings in the areas of hazard identification and emergency response [12].

The objective of this study was to determine the safety practices among laboratory technicians and assess their perceptions of the existing system of safety.

## Materials and methods

This cross-sectional and descriptive study was carried out in the Central Laboratory, Sri Ramaswamy Memorial (SRM) Medical College Hospital and Research Centre, Kattankulathur, Chennai, India, from October to November 2022. Institutional ethical committee approval was obtained (SRMIEC-ST0922-192).

All laboratory technicians who gave consent were included in the study. Among the lab technicians, 40.67% (n = 61) had a Diploma in Medical Laboratory Technology, 52.67% (n = 79) BSc in Medical Laboratory Technology, 5.33% (n = 8) MSc in Medical Laboratory Technology, 1.33% (n = 2) PhD in Medical Laboratory Technology.

The technicians who were not willing to take part and did not give consent were excluded from the study.

The sample size is 150, based on a previous study [13]: n = Z²pq/d2, where p = 38.7, q = 61.3, and d = 7.7. Substituting the values in the formula gives us: n = [(1.96)² x 38.7 x 61.3] / (7.7)² = 150.

A questionnaire was prepared using Google Forms and sent to laboratory technicians’ official email addresses and through WhatsApp, containing the following items: (A) demographic data, name, age, and gender (questions 1-2); (B) professional data, department, experience, and education (questions 3-5); and (C) occupational safety practices among laboratory employees (questions 7-15) and occupational safety perceptions related to job responsibilities (questions 16-22), related to the work environment (questions 23-30), related to patient safety (questions 31-44), related to routine work and communication (Questions 45-47), and related to human resources (questions 48-50).

The validity of the questionnaire was statistically analyzed by internal and external experts.

A pilot study was conducted to assess reliability (10% of the sample size, 15 participants) but was not included in the main study. Reliability scores (Cronbach’s alpha) of the items in the questionnaire were calculated using IBM SPSS Statistics for Windows, Version 23.0 (Released 2015; IBM Corp., Armonk, New York, United States). The questionnaire contained 44 questions. The Cronbach’s alpha for the questions was 0.689. The Cronbach’s alpha measures internal consistencies. A score >0.8 to 1.0 is very reliable, >0.6 to 0.8 is reliable, >0.4 to 0.6 is quite reliable, >0.2 to 0.4 is rather reliable, and 0.0-0.2 is less reliable.

Further, these questions on safety practices and perceptions were coded into good, fair, and poor categories for statistical analysis (Table 1).

**Table 1:** Coding for statistical analysis

S. no.	Study variables	Coding categories
1.	Safety practices	Good, score of 7-9; fair, score of 4-6; poor, score of 0-3
2.	Perception related to job responsibilities	Good, score of 5-6; fair, score of 3-4; poor, score of 0-2
3.	Perception related to the work environment	Good, score of 7-8; fair, score of 4-6; poor, score of 0-3
4.	Perception related to patient safety	Good, score of 9-12; fair, score of 5-8; poor, score of 0-4
5.	Perception related to routine work and communication	Good, score of 5-6; fair, score of 3-4; poor, score of 0-2
6.	Perception related to human resources	Good, score of 3; fair, score of 2; poor, score of 1
7.	Overall perception score	Good, score > 31; poor, score < 31

Statistical analysis was performed using Microsoft Excel 2010 (Microsoft Corp., Redmond, WA) and IBM SPSS Statistics for Windows, Version 23.0 (Released 2015; IBM Corp., Armonk, New York, United States). A descriptive analysis was done. Both frequency and percentage were used to present the categorical variables. A chi-square test was used to determine the relationship between two categorical variables.

## Results

Laboratory technicians working in the SRM Medical College Hospital and Research Centre, Kattankulathur, Tamil Nadu, took part in the present study. The completed questionnaire from the technicians was submitted for analysis (Appendices).

In this study, there were 99 female and 51 male participants. Seventy-eight percent of the participants belong to the 21-30 age group. Fifty-eight percent of the participants work in the pathology department, 18.7% in the biochemistry department, 16% in the microbiology department, and 7.3% in the blood bank department. Of the participants, 76.7% (n = 115) had less than 10 years of experience, and 23.3% (n = 35) had more than 10 years of experience. The demographic and professional data of the participants are depicted in Table 2.

**Table 2:** Demographic and professional details (n = 150)

Variable	Frequency	%
Age (years)		
21-30	117	78.0
31-40	20	13.3
>40	13	8.7
Gender		
Male	51	34.0
Female	99	66.0
Experience (years)		
<5	115	76.7
>5	35	23.3
Department		
Biochemistry	28	18.7
Blood bank	11	7.3
Microbiology	24	16.0
Pathology	87	58.0

In this study, the perception related to job responsibilities was graded as good in 97.9% (n = 147). The perception related to the work environment showed good safety practices in 17.3% (n = 26), fair safety practices in 51.4% (n = 77), and poor safety practices in 31.3% (n = 47). In this study, 97.9% (n = 150) of the technicians had affirmative responses regarding perceptions related to patient safety. The perception related to routine work and communication was good among 96% (n = 144) of the technicians and fair among 4% (n = 6) of the technicians. In the perceptions related to human resources, 81.3% (n = 122) had given a positive response. In the safety practices among laboratory technicians, it was noted that 62% (n = 93) do not wash their hands before wearing gloves, and 20% (n = 30) consume food and beverages during work hours. In the responses to perceptions related to job responsibilities, 50.7% (n = 76) of the lab technicians consider that noise in the workplace causes a disturbance, and 47.3% (n = 71) consider that infrequent cleaning of air conditioners (ACs) cause health issues. Seventy-two percent (n = 108) of the laboratory technicians consider that they are at greater risk of infection due to inadequate personal protective equipment (PPE) and 56.7% (n = 85) due to inadequate knowledge of PPE. The responses to the rest of the questions pertaining to safety practices and perceptions were between 82 and 100% (Table 3).

**Table 3:** Mean score for details of safety practices and perceptions (n = 150) AC: air conditioner, PPE: personal protective equipment, TAT: time and temperature.

S. no.	Question	Yes	%
1	Wearing gloves everyday as a routine	145	96.7
2	Removing gloves while working on a computer/mobile phone	125	83.3
3	Washing hands before wearing gloves	57	38.0
4	Washing hands after removing gloves	149	99.3
5	Changing gloves when they are torn or untidy	142	94.7
6	Wearing protective aprons in the lab	145	96.7
7	Consuming food and beverages during work	30	20.0
8	Maintaining clean, short nails	150	100.0
9	Attended orientation program to perform my job	131	87.3
10	Satisfied with the work they do	148	98.7
11	Treated with respect in the workplace	143	95.3
12	Aware of duties and responsibilities of my job	149	99.3
13	Feels the job skills are evaluated satisfactorily	146	97.3
14	Feels the working atmosphere is good	144	96.0
15	Feels superiors are providing good guidance/support	149	99.3
16	Feels noise in the workplace is causing disturbance	76	50.7
17	Health issues due to infrequent cleaning of ACs	71	47.3
18	Quality of cleaning the lab/workplace is adequate	139	92.7
19	Lab technicians are at great risk of infection	108	72.0
20	Great risk of infection due to inadequate PPE	91	60.7
21	Great risk of infection due to inadequate knowledge of PPE	85	56.7
22	Workers have sufficient private space	123	82.0
23	Workspace fixtures and fittings are sufficient	135	90.0
24	Important to always use gloves when handling bio-samples	147	98.0
25	Aware of careful handling of samples during transport and processing	148	98.7
26	TAT must be adhered to while transporting and processing samples	130	86.7
27	Analytical processes done manually have high chances of error	132	88.0
28	Important to determine labelled requests without barcodes	148	98.7
29	Aware of procedures to ensure correct identification of samples	145	96.7
30	Mistakes related to obtaining samples should be recorded	145	96.7
31	Mistakes related to patient identification should be notified	144	96.0
32	All laboratory errors are analyzed, though they are not harmful	149	99.3
33	All the incidents during the processing of samples should be notified	149	99.3
34	Understand the need for compiling records concerning patient safety	148	98.7
35	Lost or missed reports are traced and recorded and action is taken	147	98.0
36	Circumstances affecting the patient safety to be communicated	141	94.0
37	Aware of reporting and recording procedure for near-critical results	147	98.0
38	When an error is reported, investigating the problem is important	148	98.7
39	Mistakes detected in the workplace are criticized in a constructive manner	145	96.7
40	Satisfied that suggestions are received by the supervisors	146	97.3
41	Satisfied that suggestions are responded to	145	96.7
42	Sufficient human resources to deal with the workload	131	87.3
43	Replacements are made for staff on medical leave or on holiday	137	91.3
44	Patient’s samples processed are proportionate to human resources	138	92.0

The relationship between safety practices with respect to demographic and professional data was analyzed. It revealed that age, gender, workplace, and experience did not have statistical significance (Table 4).

**Table 4:** Safety practices and demographic and professional details (n = 150)

Variable	Safety practices				Odds ratio (95% CI)	χ^2 ^value (p-value)
	Good (n = 143)		Fair (n = 7)			
Age						
21-30 (n = 117)	113	79.0	4	57.1	1	0.804 (0.369)
>30 (n = 33)	30	21.0	3	42.9	2.8 (0.6-13.3)	
Gender						
Male (n = 51)	47	32.9	4	57.1	2.7 (0.6-12.7)	0.837 (0.360)
Female (n = 99)	96	67.1	3	42.9	1	
Experience (years)						
<5 (n = 115)	112	78.3	3	42.9	1	2.918 (0.087)
>5 (n = 35)	31	21.7	4	57.1	4.8 (1.0-22.6)	
Department						
Biochemistry (n = 28)	25	17.5	3	42.9	-	0. 312
Blood bank (n = 11)	11	7.7	0	0.0		
Microbiology (n = 24)	24	16.8	0	0.0		
Pathology (n = 87)	83	58.0	4	57.1		

Safety practices in relation to safety perceptions among the study participants showed that there was statistical significance with respect to human resources, whereas there was no statistical significance in association with employee perceptions related to job responsibilities, work environment, patient safety, and routine work and communication (Table 5).

**Table 5:** Safety practices and perceptions among the study participants (n = 150)

Perception	Safety practices				Odds ratio (95% CI)	χ^2 ^value (p-value)
	Good (n = 143)		Fair (n = 7)			
Related to job responsibilities						
Good (n = 147)	141	98.6	6	85.7	1	0.990 (0.319)
Fair/poor (n = 3)	2	1.4	1	14.3	11.8 (0.9-148.4)	
Related to the work environment						
Good (n = 26)	25	17.5	1	14.3	1	0.085 (0.769)
Fair/poor (n = 124)	118	82.5	6	85.7	1.3 (0.1-11.0)	
Related to patient safety						
Good (n = 147)	140	97.9	7	100.0	1	1.000
Fair/poor (n = 3)	3	2.1	0	0.0	2.7 (0.1-56.7)	
Related to routine work and communication						
Good (n = 144)	138	96.5	6	85.7	1	0.189 (0.663)
Fair/poor (n = 06)	5	3.5	1	14.3	4.6 (0.5-45.8)	
Related to human resources						
Good (n = 122)	119	83.2	3	42.9	1	4.748 (0.029)*
Fair/poor (n = 28)	24	16.8	4	57.1	6.6 (1.4-31.4)	

## Discussion

Nations are influenced differently by the risks and unfavorable occurrences associated with healthcare systems. Although the issue may initially appear to be one of personal accountability on the part of the employees, it still relates to the system. In light of this, experts have suggested that research nations should focus on fostering and developing a safety-oriented culture. Also, organizations should be aware of the importance of transforming and improving their safety culture [14,15]. One of the strategies for raising the standards of healthcare is to regularly assess the safety climate. This allows for the identification of aspects that require more attention and collaboration to create a safety culture that is conducive to continuous improvement. It is claimed that a bad safety culture, where the punitive culture, centered on blaming someone for the mishaps, prevails, might create an unstable workplace, making it more likely that adverse events will occur [16]. Therefore, it is essential to assess the problem in our area of expertise, the clinical laboratory, and to come up with possible solutions.

Self-report surveys are commonly employed to examine health-related behaviors, which are useful tools for observational studies and for evaluating educational interventions [17]. The present study has a broader range of questions for a deeper understanding and interrelationship to determine the variation in safety practices and perceptions related to multiple factors among various working groups and to identify the factors that interfere with the safety of laboratory healthcare workers and in improving the organizational culture and healthcare quality. Of the 150 participants in the study, nearly two-thirds of the staff were female lab technicians. A similar observation was made by Giménez-Marín et al., who mentioned that due to social and cultural changes, women are noted to be the predominant working population among paramedical healthcare workers [18].

Demographic profile

This study was conducted in a 1635-bed tertiary care hospital with a central lab and many side labs in the ward for prompt disposal of services. Seventy-eight percent of the study participants belong to the 21-30 age group, with 22% of the working population having less than 10 years of experience. In contrast, the study conducted by Carvalho et al. in Brazil and Giménez-Marín et al. in Spain observed that 47.2% of the participants belonged to the 30-59 age group, with more than 11 years of experience [16,18]. The authors consider it a positive factor of safety culture, as it reflects the employees’ stability, common shared background, and learning experience. A “safety culture” is an organizational culture that places a high level of importance on safety attitudes and behaviors [4].

Safety practices

In this study, 96.7% of lab technicians were wearing gloves as a daily routine, and 83.3% of them removed gloves while using a computer or phone. Ninety-nine percent wash their hands after removing gloves, and 94.7% change their gloves when they are torn or untidy. In the lab, 96.7% wear protective aprons, and 100% maintain clean, short nails. These measures indicate that the employees are strongly adhering to the safety practices. In contrast, Dukic et al. describe low safety practices as only 12.5% and 61.3% of the participants always wear protective clothing and gloves, respectively [19]. The two main justifications given for not wearing gloves are because of habits and the fact that they interfere with their work. A small but worrying number of participants in the study by Dukic et al. mentioned that gloves are not readily available in their workplace. Similar observations were made by Simundic et al., Main et al., Akhter et al., Abdulraheem et al., and Alice et al. [20-24].

In a study about the adoption of suggested personal protective practices among 1268 Canadian laboratory employees, Main and colleagues presented their findings [21]. They noted a high degree of noncompliance with precautions: 41% of the participants do not always use gloves when handling body fluids, and 62% of the participants do not regularly wear gloves when handling blood and blood products [21]. Another team of authors polled the medical technologists working in their institution’s Department of Pathology and Laboratory Medicine in Saudi Arabia. The findings revealed that while a remarkable 92% of the participants fully complied with the recommendation to wear gloves at all times when handling blood and blood products, only 61% had practiced hand hygiene after the removal of gloves [22]. Phukan reported that laboratory technicians and nurses in South India do not adhere to safety behaviors, despite being well aware of the need to wash their hands and wear gloves (87.5% and 88.3%, respectively). Only 14.2% of the laboratory technicians wash their hands correctly, and only 35.0% use gloves [25].

Few centers insist on washing hands before reaching for gloves, as it avoids the contamination of other gloves in the box [26]. In the present study, only 38% performed handwashing before wearing gloves. Rock et al. mentioned that hand hygiene before donning gloves is time-consuming and unnecessary as it does not reduce the transmission of pathogens [27]. In this study, 20% of the lab employees consume food and beverages during work hours. Despite the fact that eating and drinking are absolutely prohibited at work, the findings of Dukic et al. show that, respectively, 46.1% and 36.4% of people do so [19,28]. Similar conclusions were noted in a Turkish study, where 38.3% of the participants consumed food or beverages while in the lab [29]. In Pakistan, however, the majority of labs lack a defined location for the same [30].

Perceptions related to job responsibilities

Of the laboratory technicians, 87.3% had attended an orientation program in relation to their job, 98.7% were satisfied with the work they do, 95.3% perceive that they are treated with respect in the workplace, 99.3% were aware of their duties and responsibilities, 97.3% consider their job skills were evaluated satisfactorily, and 92.7% feel that the working atmosphere is good. Ninety-six percent consider that their superiors are providing guidance and support. A similar observation was reported by Giménez-Marín et al. These results reflect the laboratory management and organization, with certain items signifying leadership, attitudes, and emotions crucial to a patient safety culture [18].

Perceptions related to the work environment

Two questions about the work environment received a lot of negative feedback, noisy surroundings (50.7%) and the poor maintenance of the AC (47.3%), despite being an inevitable outcome, as they have an impact on the work environment. A similar observation was made by Carvalho et al. [16]. Noise poses a significant risk as identified by healthcare professionals, according to Ilgar and Vehid et al. [31,32]. Other sources of potential workplace hazards that healthcare workers are exposed to include medical waste, electrical equipment, noise, and faulty AC systems [33]. Seventy-two percent of the lab employees consider that they are at greater risk of infection due to inadequate PPE while 56.7% due to inadequate knowledge of PPE. This is an important issue to be addressed, as it threatens the safety of the laboratory employees. Similarly, Ahmad et al. reported that 65% of the laboratory technicians in Karachi did not wear any kind of PPE and proposed basic training programs for raising laboratory personnel’s awareness of biosafety standards [30].

Perceptions related to patient safety

Over two-thirds of medical decisions are based on the results of diagnostic tests, making clinical laboratories, which are distinguished by their well-defined processes, pioneers in advancing analytical quality and a crucial link in the healthcare chain [34]. In the context of a clinical laboratory, the term “patient safety culture” refers to both the proactive involvement of the staff (safe systems, behavior management) and the firm’s acknowledged commitment to patient safety as demonstrated by the staff’s perceptions, skills, and attitudes. It is well known that the majority of errors, and those that are most important to patient safety, occur in situations outside the laboratory itself, aside from decisions pertaining to the structure of the healthcare organization. The mistakes frequently endanger patient safety by degrading the analytical report’s validity and usefulness [35]. In the present study, between 86.7% and 98% of the lab employees had good patient safety perceptions pertaining to patient identification, careful transport, processing of bio-samples, turnaround time, recording, and notification of errors. A similar observation was made by Giménez-Marín et al., who remarked that professional groups, due to their engagement in the maintenance of quality policies, heads of services, or sections, report the greatest impressions of patient safety, far higher than that of various groups, with administrative employees at the other end of the spectrum [18].

The factors that vary significantly within the same institution have to be considered, depending on the research participants and timing, and therefore, methodological rigor, as followed in this study, is necessary to provide accurate and reliable results. On the other hand, the positive sign of the institution’s adherence to patient safety protocols with a more favorable outcome cannot be undermined [35]. In affluent nations like 65.9% in Ireland, 78% in Sweden, and 72% in the United States, the safety climate has had favorable results [36-38].

Perceptions related to routine work and communication

It represents teamwork, the harmony or synchrony that enhances the goal of accomplishing quality healthcare. In the present study, the frequency reported ranges between 94% and 100%. A favorable safety atmosphere was appreciated by the lab employees, which is found to be higher than the findings from a Brazilian research (59.1%) as well as those from Chinese studies (48.9%) [16,39].

Perceptions related to human resources

TThe motivation of employees and indeed the outcomes attained are both known to be significantly impacted by the organizational atmosphere. It has a significant effect on the workforce and represents high-risk areas that need to be targeted to maintain patient safety [40-44]. In the present study, the affirmative response rate pertaining to human resource availability and management was between 87% and 92%, which indicates that this factor does not affect the safety practices of the lab employees. Similarly, Giménez-Marín et al. also observed a good response rate of about 97%-98% in the hospitals in Spain [18].

Relation of safety practices with demographic profile

The relationship between the safety practices and the demographic profile revealed that age, gender, experience, and place of work did not have statistical significance for safety practices. A similar observation was made by Alshalani and Salama [45].

Relation of safety practices with safety perception

In the present study, safety practices in relation to safety perceptions were statistically significant with respect to human resources. However, there was no statistical significance with respect to job responsibilities, work environment, patient safety, and routine work or communication. However, Carvalho et al. observed a correlation between working circumstances, managerial perspective, and job satisfaction [16]. In a nutshell, the study’s findings paved the way for recognizing the gaps and strengths of the organization in the development of high-quality healthcare and patient safety in clinical laboratories.

Strengths and limitations of the study

The strengths of the study include multidisciplinary input and a broad range of questions covering multiple dimensions of employee safety and satisfaction, which have an impact on quality healthcare and patient safety. The study limitations include respondent bias, which could not be accounted for because this is a questionnaire-based survey. As this is a single-center survey, the outcomes of the study may not be extrapolated. Though there is a most encouraging finding in this study due to the institution’s patient safety protocols, it had to be assessed from time to time as the factors may vary significantly between different institutions or even within the same institution depending on the research participants and timing. Therefore, methodological rigor is necessary to provide accurate and reliable results, to generalize the results all over the country. Future multicenter studies of this nature should be conducted in India to understand how sociocultural factors affect the standard of healthcare delivery, to ascertain the safety perceptions and practices in accredited and non-accredited laboratories, and to determine the safety perceptions and practices in the various regions of the country.

## Conclusions

Overall, lab employees have high awareness and good adherence to safety practices. Despite the institution’s laboratory following established quality norms and procedures, this study was helpful in identifying areas that needed attention and may be addressed by staff interaction and ongoing training programs on safety. The current study concludes that frequent measurement of the safety climate at regular intervals is one of the strategies to improve the culture of the organization, motivate the workforce, and improve the quality of health services because it enables the identification of the aspects that require more attention and collaboration on quality to create a safety culture that encourages continuous improvement. Future multicenter studies of this nature should be conducted in India to understand how sociocultural factors affect the standard of healthcare delivery, to ascertain the safety perceptions and practices in accredited and nonaccredited laboratories, and to determine the safety perceptions and practices in the various regions of the country.

## Appendices

Lab employee safety questionnaire

Demographic Data

1. Age o 21-30 o 31-40 o 41-50 o 51-60 o >60

2. Gender o Male o Female

Professional Data

3. Department o Biochemistry o Pathology o Microbiology o Blood bank

4. Experience o <5 years o 6-10 years o 11-15 years o 16-20 years o >20 years

5. Education o D MLT o BSc MLT o MSc MLT o PhD

Occupational Safety Practices

6. I wear gloves everyday as a routine

o Yes  

o No

7. I remove the gloves while working on computer/mobile phone

o Yes  

o No

8. I wash my hands before wearing gloves

o Yes  

o No

9. I wash my hands after removing gloves

o Yes  

o No

10. I change my gloves when they are torn or untidy

o Yes  

o No

11. I wear protective aprons in the lab

o Yes  

o No

12. I consume food and beverages in during work

o Yes  

o No

13. I maintain clean, short nails

o Yes  

o No

14. I have received refresher training (orientation) course to perform my job

o Yes  

o No

Occupational Safety Perception (Related to Job Responsibilities)

15. I am satisfied with the work I do

o Yes  

o No

16. I am treated with respect in the workplace

o Yes  

o No

17. I am aware of the duties and responsibilities of my job

o Yes  

o No

18. I have my own identity within the organization

o Yes  

o No

19. I consider that my acquisition of job skills is evaluated satisfactorily

o Yes  

o No

20. I consider that the working atmosphere is good

o Yes  

o No

21.  I consider that my superiors are providing good management and support

o Yes  

o No

Occupational Safety Perception (Related to the Work Environment)

22. I consider the noise from equipments in the workplace is causing disturbance

o Yes  

o No

23. I have frequent health issues due to infrequent cleaning of air conditioners

o Yes  

o No

24. I consider the quality of cleaning in the lab is adequate

o Yes  

o No

25. I consider lab technicians are at great risk of infection

o Yes  

o No

26. I consider that I am at greater risk of infection due to inadequate personal protective equipments

o Yes  

o No

27. I consider that I am at greater risk of infection due to inadequate knowledge of using appropriate protective equipments

o Yes  

o No

28. I consider that workers have sufficient private space

o Yes  

o No

29. The amount of workspace available is sufficient

o Yes  

o No

30. I consider the ergonomics of workspace fixtures and fittings are sufficient

o Yes  

o No

Occupational Safety Perception (Related to Patient Safety)

31. I consider that it is important to use gloves always when handling biological samples

o Yes

o No

32. I am aware that during the transport of patient samples (IP/OP/ICU), the time and temperature should be maintained

o Yes  

o No

33. I am aware that the analytical processes that are performed manually have high chances of error, which is detrimental to patient safety

o Yes  

o No

34. I consider that it is important to determine the number of analytic requests that are labelled but lack patient identification barcode

o Yes  

o No

35. I am aware of the procedures and actions to ensure the correct identification of patient’s samples

o Yes  

o No

36. I am aware that mistakes related to obtaining samples should be recorded

o Yes  

o No

37. I am aware that mistakes related to patient identification  should be notified

o Yes  

o No

38. I am aware that all laboratory errors are analyzed, even if they pose no potential harm to the patient

o Yes  

o No

39. I am aware that all the incidents during the processing of the samples should be notified

o Yes  

o No

40. I understand why records concerning patient safety are compiled and kept

o Yes  

o No

41. I am aware that the lost or missed reports are traced and recorded and action is taken

o Yes  

o No

42. I feel that their mistakes are used against them

o Yes  

o No

43. I am satisfied that my suggestions are received by my seniors

o Yes  

o No

44. I am satisfied that my suggestions are responded to

o Yes  

o No

Occupational Safety Perception (Related to Routine Work and Communication)

45. I consider that it is important to communicate the circumstances whenever anything is affecting patient safety.

o Yes  

o No

46. I consider that when an error is reported, the investigation of the problem is important.

o Yes  

o No

47. I am aware of the procedure of reporting and recording correctly the near-critical results in my unit.

o Yes  

o No

Occupational Safety Perception (Related to Human Resources)

48. There are sufficient human resources to deal with the workload

o Yes

o No

49. Replacements are made for staff on medical leave or on holiday

o Yes  

o No

50. The number of patient’s samples processed is appropriate to the human resource available

o Yes  

o No
